# LGL Clonal Expansion and Unexplained Cytopenia: Two Clues Don’t Make an Evidence

**DOI:** 10.3390/cancers14215236

**Published:** 2022-10-25

**Authors:** Giulia Calabretto, Enrico Attardi, Carmelo Gurnari, Gianpietro Semenzato, Maria Teresa Voso, Renato Zambello

**Affiliations:** 1 Department of Medicine, Padua University School of Medicine, Hematology Division, 35129 Padua, Italy; 2 Veneto Institute of Molecular Medicine (VIMM), 35129 Padua, Italy; 3 Department of Biomedicine and Prevention, University of Rome Tor Vergata, 00133 Rome, Italy; 4 Translational Hematology and Oncology Research Department, Taussig Cancer Center, Cleveland Clinic, Cleveland, OH 44106, USA

**Keywords:** large granular lymphocytes, cytopenias, clonality, LGLL, bone marrow failure syndromes

## Abstract

**Simple Summary:**

The clonal expansions of large granular lymphocytes are frequently detected in a wide spectrum of hematological and immune diseases. This clinical overlap, especially in patients with unexplained cytopenia, makes their diagnostic classification particularly challenging. Herein, we aim to elucidate the boundaries between LGL leukemia (LGLL) and LGL clonal expansions. We also discuss the relevance of LGL clone detection in the diagnostic algorithm, as they might acquire different pathogenetic roles according to the diverse clinical setting.

**Abstract:**

Clonal expansions of large granular lymphocytes (LGL) have been reported in a wide spectrum of conditions, with LGL leukemia (LGLL) being the most extreme. However, the boundaries between LGLL and LGL clones are often subtle, and both conditions can be detected in several clinical scenarios, particularly in patients with cytopenias. The intricate overlap of LGL clonal expansion with other disease entities characterized by unexplained cytopenias makes their classification challenging. Indeed, precisely assigning whether cytopenias might be related to inadequate hematopoiesis (i.e., LGL as a marginal finding) rather than immune-mediated mechanisms (i.e., LGLL) is far from being an easy task. As LGL clones acquire different pathogenetic roles and relevance according to their diverse clinical settings, their detection in the landscape of bone marrow failures and myeloid neoplasms has recently raised growing clinical interest. In this regard, the current availability of different diagnostic techniques, including next generation sequencing, shed light on the relationship between LGL clones and cytopenias, paving the way towards a better disease classification for precision medicine treatments. Herein, we discuss the clinical relevance of LGL clones in the diagnostic algorithm to be followed in patients presenting with cytopenias, offering a foundation for rational management approaches.

## 1. Introduction

Large granular lymphocytes (LGLs) functionally represent the cytotoxic arm of the immune system. Their physiological activation and expansion is generally transient, polyclonal, and self-limited. However, chronic antigenic pressure or acquired molecular alterations might promote the selection of an LGL clone as part of a normal immune response, leading to its aberrant proliferation, eventually resulting in full-blown LGL leukemia (LGLL) [1,2]. Looking beyond the boundaries of LGLL disease, LGL clonal expansions have been reported in a wide spectrum of conditions, including autoimmune disorders, immunodeficiencies, and clonal myeloid diseases [3,4,5]. Cytopenias are the narrative thread and are the most common clinical presentation of almost all of these conditions, highlighting the need to properly address differential diagnosis [6]. 

While in LGLL the cytotoxic clone is responsible for the presence of cytopenias, whose severity represents the main indication of treatment start [7], the potential contribution of LGL clonal expansions in the above-mentioned conditions is still unclear. This might be part of an immune surveillance reaction, as well as the result of a common etiopathogenesis, also contributing to disease progression. However, in most cases, the peculiar concurrence of LGL clonal expansion with other disease entities still has a controversial impact on their prognosis and outcome. Moreover, a recent study highlighted a high degree of overlap between leukemic and non-leukemic T cell receptor (TCR) repertoires via possible common target antigens, further expanding the pathogenic routes leading to disease manifestations [8]. In such a scenario, the detection of LGL clonal expansions has recently raised increased interest, as the precise determination of its clinical significance is oftentimes unclear. 

Herein, we focus on the relevance of LGL clones with respect to diagnostic orientations, by stressing the role of a multi-step approach based on the investigation of discrete biological alterations and according to the diverse clinical settings. In particular, we critically discuss some hints that could help to properly address differential diagnoses in the presence of LGL clones and cytopenias, two clues that could be the sentry of a variety of underlying pathological conditions.

## 2. Detection and Characterization of LGL Clones

LGLs account for approximately 10–15% of peripheral blood mononuclear cells (PBMC), with an absolute count ranging from 0.2 to 0.4 × 10^9^ LGL/L. These types of cells can be CD3+ T cytotoxic lymphocytes or CD3- Natural Killer (NK) cells, which are two distinct but functionally related cell types, with cytotoxic properties [9]. 

Physiological LGL expansions are generally polyclonal and related to pathogenic *noxae* or antigenic stimuli. Upon antigen clearance, engaged cells undergo activation-induced cell death (AICD), which is a central mechanism to maintain immune homeostasis. Conversely, a chronic antigenic stimulation, as well as additional cooperating events (i.e., inflammation, impairment of AICD, genetic lesions, and constitutive activation of pro-survival pathways), might promote the selection of an immunodominant LGL clone, which persists and proliferates over time [10].

**Figure 1:**
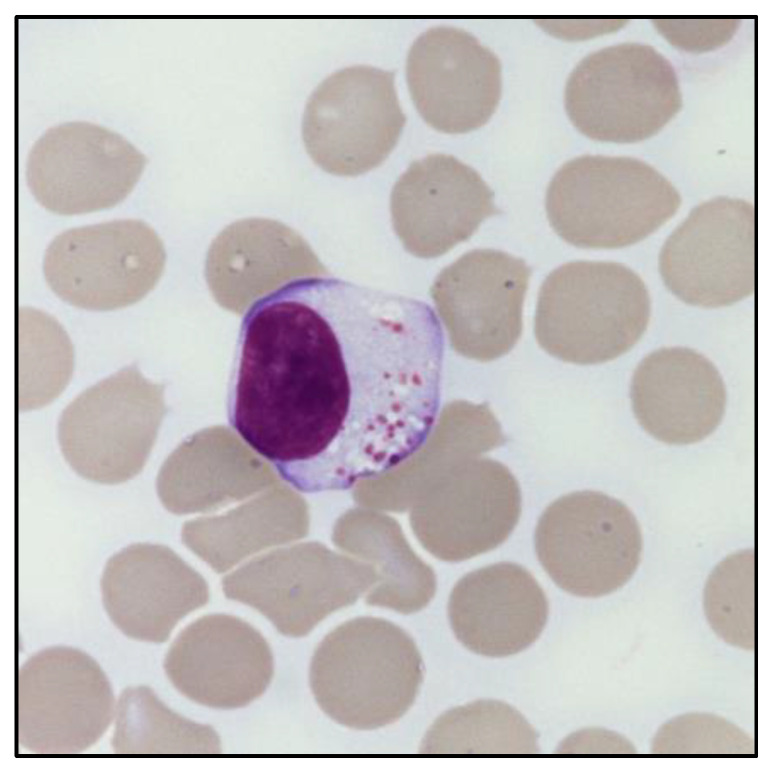
Morphology of a large granular lymphocyte. May–Grunwald Giemsa staining of large granular lymphocytes in the PB of a patient with LGL leukemia. Magnification: optical microscope 1000×.

The presence of an expanded LGL population can be defined based on its absolute count (i.e., greater than 0.5 × 10^9^/L). In this regard, peripheral blood (PB) smear can provide an immediate evaluation of the LGL percentage in relation to the total lymphocytes, which is useful to estimate the absolute LGL count. Morphologically, LGL are characterized by a size (≃15–18 μm) larger than the other circulating lymphocytes [9]. In particular, these cells have a round or reniform nucleus and an abundant pale cytoplasm with characteristic azurophilic granules, containing cytolytic components such as perforin and granzymes. Of note, clonal LGL do not show any morphological difference compared to polyclonal LGL, nor any distinctive signs of clonality (Figure 1). The only exception exists in the cytological atypia observed in leukemic NK cells of patients diagnosed with Aggressive NK cell leukemia (ANKL) [11].

Flow cytometry (FC) furthers the information obtained by PB smear, helping in the first discrimination between CD3+ T-LGL and CD3− NK cells. In detail, T-LGLs are terminal-effector memory T cells (TEMRA), which typically show a CD3+, CD4−, CD5^dim^, CD8+, CD27−, CD28−, CD45RA+, CD45RO−, CD57±, CD62L−, CCR7−, CD122+ phenotype. These can be equipped with an αβ-TCR or, less frequently, with a γδ-TCR. Conversely, NK cells are devoid of TCR and exhibit a CD2+, sCD3−, CD3ε+, CD4−, CD8+, CD16+, CD56+, CD57± phenotype. The aberrant expression of NK cell markers and receptors, including Killer Immunoglobulin-like Receptors (KIR) and the CD94/NKG2 heterodimers, is often seen in both clonal T- and NK-LGL proliferations [12,13]. 

In the setting of an expanded LGL population, clonality should be assessed to differentiate reactive from clonal expansions. Molecular-based approaches and FC are currently the gold standard [1]. The clonality of the T-cell lineage can be detected by polymerase chain reaction (PCR), evaluating the TCR repertoire [14]. In addition, next generation sequencing (NGS) of the antigen-binding region (i.e., the complementarity determining region 3, CDR3) of TCR provides information regarding the specific CDR3 sequences and the size of the immunodominant clonotype [15]. Flow cytometer analysis of the TCR Vβ expression offers an additional and rapid approach to assess the preferential expression of one TCR-Vβ segment [16]. Remarkably, recent advances in high-sensitive next-generation FC are now extending the current diagnostic possibilities. In this regard, evaluation of the constant region 1 of the T-cell receptor chain (TCRBC1) by FC has recently been proposed for assessing T clonality [17].

The identification of clonality in NK cell proliferations is more difficult, given the absence of TCR as a possible marker. An analysis of the restriction fragment length polymorphism (RFLP) has been used in a few cases, but this evaluation is gender-related. Otherwise, evidence of a restricted pattern of KIR or NKG2 expression, provided by FC, is accepted as a surrogate marker of clonal expansion [18,19,20].

## 3. Large Granular Lymphocytes Leukemia: From Phenotypic to Genetic Heterogeneity

According to well-defined criteria, the chronic expansion of a clonal LGL population in the peripheral blood meets the diagnosis of LGL leukemia (LGLL) [1]. Based on the recently published fifth edition of the World Health Organization (WHO) Classification of Haematolymphoid Tumours (Lymphoid Neoplasms), LGLL is classified within the Mature T-cell and NK-cell neoplasms section [20]. 

The disease encompasses a remarkable clinical, phenotypic, and genetic heterogeneity. According to the immunophenotype of the leukemic clone, three main subtypes can be recognized: T-large granular lymphocytic leukemia (T-LGLL), NK-large granular lymphocytic leukemia (NK-LGLL, previously listed as a provisional entity, i.e., Chronic lymphoproliferative disorder of NK cells) [21], and aggressive NK-cell leukemia (ANKL). 

T-LGLL and NK-LGLL are the most frequent forms of the disease (85% and 10% of cases, respectively) [13], while ANKL is a rare variant (accounting for approximately 5% of cases), typically seen in Asian populations and characterized by EBV positivity [22]. 

Within T-LGLL, additional immunophenotypic subtypes can be identified [20]. Based on the type of TCR, two variants (Tαβ- and Tγδ-LGLL) can be distinguished. Moreover, beyond the canonical CD4−/CD8+ T-LGLs expansion (CD8+ T-LGLL), a CD4+/CD8^neg/dim^ variant (CD4+ T-LGLL) has been described in ~30% of cases [23,24]. This distinction does not represent just a formal classification, as accumulating evidence is showing that these subsets are characterized by different biological and molecular features [24,25,26]. Several molecular alterations contribute to the survival and proliferation of the expanded LGL clone [1], with some of them being a signature of a precise disease subtype [27]. Among these, *STAT3* and *STAT5b* hyperactivating mutations represent the main genetic lesions of leukemic LGL, and are particularly relevant because of their association with phenotype, clinical manifestations, and disease prognosis [26,28]. In detail, *STAT3* mutations are common in CD8+ Tαβ-LGLL and Tγδ-LGLL (Vδ2-/CD56-) subsets and are associated with cytopenias and reduced overall survival [24,29,30]. Conversely, *STAT5b* mutations are more typically present in CD4+ T-LGLL and have also recently been found also in Tγδ-LGLL (Vδ2+/CD56+) [30,31]. In contrast with *STAT3*, *STAT5b* mutations are generally linked to an indolent course, similar to that of *STAT3* wild-type cases [30]. Rarely, *STAT5b* mutations can be found in association with the peculiar CD3+/CD8+/CD4−/CD16−/CD57−/CD56+ phenotype and, in this case, are associated with an aggressive T-LGLL variant characterized by a poor prognosis [32]. In the NK-LGLL subtype, *STAT3* mutations usually occur in LGL clones expressing CD16^high^/CD56^dim/neg^/CD57− and clinically manifest with severe neutropenia [33], whereas *STAT5b* mutations have rarely been detected [34].

## 4. T-LGLL and NK-LGLL Diagnosis: Stringent Criteria and Recommended Analyses

A diagnosis of LGLL needs to be considered in the context of an integrated approach, including the phenotypic and molecular characterization of the LGL clone and an evaluation of the clinical context. 

A temporal criterion with a chronic LGL expansion, generally lasting more than 6 months since its initial detection, is essential to first exclude reactive conditions. Of note, a lymphocytosis of at least 2 × 10^9^ LGL/L was historically mandatory for fulfilling a LGLL diagnosis. Based on the acquired experience, a reduced threshold of 0.5 × 10^9^ LGL/L in PB is now commonly accepted [20], if other criteria for the disease are present (evidence of clonality, typical immunophenotypic pattern, and clinical features). Proof of clonality can be obtained, as described above, according to the proliferating cell type (T- or NK-LGL). 

FC is a milestone in the diagnostic work-up of LGLL. As aforementioned, patients exhibit a high phenotypic heterogeneity, albeit with a strong correlation between some immunophenotypic features and the occurrence of neutropenia (the hallmark of the disease). In detail, neutropenic T-LGLL patients are often characterized by a peculiar combination of LGL markers (i.e., CD8+/CD16+/CD56−/CD57± in the Tαβ forms or Vδ1+/CD16+/CD56−/CD57+ in the Tγδ proliferations) [29,30,31]. Similarly, neutropenic NK-LGLL patients are typically characterized by a CD16^high^/CD56^dim/neg^/CD57- phenotype [33]. In this context, FC analysis might acquire an added value, providing relevant information to precisely identify LGLL and cases requiring clone-targeting treatments. 

In some instances, the absence of a well-defined LGL lymphocytosis or evidence of clonality lead to a gray zone. The integrated evaluation of the clinical context and molecular information, provided by BM investigation and genetic analyses, could help the confirmation of a suspected diagnosis of LGLL. In particular, a diagnosis of LGLL requires the exclusion of T-cell clones of uncertain significance (T-CUS), a condition mimicking T-LGLL in terms of the immunophenotype but devoid of other clinical or laboratory features supporting a diagnosis of T-cell malignancy [35,36]. 

BM aspirate/biopsy are the mostly recommended procedures when the diagnosis is not straightforward [37]. In particular, BM morphology might help to elucidate the etiology of unexplained cytopenias, ruling out other possible differential diagnoses. The BM of LGLL patients is generally characterized by a moderate or marked hypercellularity and an interstitial lymphoid infiltration, with clusters of at least eight CD8+/TIA1+ cells or six granzyme B+ lymphocytes as a common histopathological finding [38,39]. A decrease in granulocyte precursors, combined with a left-shifted myeloid maturation, is a typical feature observed in BM of neutropenic LGLL patients. BM biopsy may also reveal the presence of fibrosis, usually increased in the BM of LGLL patients, with a grading ranging from moderate to severe in approximately 50–60% of cases, irrespective of prior treatments [40].

Cytogenetic abnormalities in LGLL are rarely investigated, because of the difficulty growing LGL in vitro. Overall, less than 10% of cases display distinct chromosomal aberrations (including 12p and 14q inversion, 5q deletion, and trisomy of chromosomes 3, 8, and 14) [13]. Conversely, molecular genetics might be useful to confirm the non-reactive nature of a LGL proliferation, as previously discussed (e.g., identification of *STATs* mutations). In particular, a novel algorithm was recently developed to classify NK-cell proliferations. The score combines FC (in terms of KIR phenotyping) and molecular profiling data with a positive predictive value of 93%. In this regard, *STAT3* and *TET2* mutations, identified with a high frequency in NK-LGLL cases (27% and 34%, respectively), have been proposed as a new diagnostic hallmark for this disease. Of note, these genetic lesions have been found to be associated with different clinical features, identifying two distinct subsets of patients. In detail, *STAT3*-mutated NK-LGLL exhibit a CD16^High^ phenotype, a decrease in hemoglobin concentration (with respect to *TET2*-mutated NK-LGLL and to reactive NK lymphocytosis), and a lower neutrophil count (compared with reactive NK expansions). In contrast, *TET2*-mutated NK-LGLL exhibit a CD16^low^ phenotype and are associated with a low platelet count and the coexistence of other hematologic malignancies [41]. In addition, somatic mutations in the C-C motif chemokine 22 (*CCL22*) gene were recently described in up to 27% of NK-LGLL patients, and could aid in discerning clonal from reactive lymphocytosis. Similarly to *TET2* mutations, these genetic lesions are associated with a CD16 ^low^ phenotype. Moreover, they have been found to be mutually exclusive with *STAT3* mutations [42]. 

Additional new mutations have also been detected in CD4+ T-LGLL [43], suggesting that the genetic landscape of the disease is evolving and mutational analyses are becoming increasingly important in distinguishing discrete disease subsets [44].

## 5. LGLL-Related Cytopenias

Although heterogeneous, the clinical course of LGLL is typically characterized by cytopenias [45]. Chronic isolated neutropenia is the most typical disease manifestation and it has been observed in up to 80% of patients, with severe neutropenia (<0.5 × 10^9^/L) characterizing up to 17–24% of cases. Neutropenic patients usually present with recurrent oral ulcerations and bacterial infections, and more rarely viral and fungal infections. Severe septic complications may also occur and represent the primary cause of disease-related death in approximately 5–10% of cases [6,9,46].

Recent evidence highlights that neutropenic T-LGLL patients share a distinctive immunophenotypic signature, which may guide their early identification. At a molecular level, a high incidence of *STAT3* mutations has been observed in both T-LGLL and NK-LGLL patients with neutropenia [28,29,30,33]. The pathogenesis of LGLL-related neutropenia has been reported as multifactorial, including immune alterations, BM infiltration/substitution, and cell-mediated cytotoxicity [47,48]. Among these, Fas-mediated apoptosis of mature neutrophils or myeloid progenitors represents one of the most commonly accepted mechanisms. Indeed, high levels of soluble FasL are detectable in the sera of neutropenic patients, as a result of a defective expression of miR-146b [27].

Beyond neutropenia, anemia (including pure red cell aplasia (PRCA)) and thrombocytopenia are other relevant manifestations [24,49,50,51,52,53]. While great efforts have been devoted to the characterization of neutropenic patients, the pathogenesis of anemia and thrombocytopenia has been less investigated.

## 6. The Boundaries between LGLL and Autoimmunity in the Pathogenesis of Cytopenia

Ideally, from a nosological standpoint, LGLL could be placed at the intersection between a clonal lymphoproliferative disorder, autoimmunity, and chronic inflammation [1,54]. In particular, the discrete association of LGLL with autoimmune disorders has emphasized the need for a refinement of diagnostic boundaries, both conditions being characterized by a spectrum of immune dysregulations and overlapping clinical manifestations, ultimately leading to the occurrence of chronic cytopenias [55].

Rheumatoid arthritis (RA) is the most frequent autoimmune condition observed in LGLL patients, and it is commonly related to T-LGLL [56]. A minority of RA patients may also develop Felty’s syndrome, a disorder characterized by the typical triad of RA, neutropenia, and splenomegaly. Remarkably, up to 40% of FS cases present a concomitant LGLL [57,58] and, in this specific instance, the integration of clinical manifestations with signs of the autoimmune disease might help in defining the nature of cytopenias [59].

Otherwise, the immune-mediated pathogenesis of cytopenia(s) in LGLL are related to the co-existence of autoimmune hemolytic anemia (AIHA), PRCA, and immune thrombocytopenia (ITP). In LGLL patients, anemia embodies, at best, the dichotomy between precursors and peripheral cell destruction. AIHA has been estimated to be the cause of anemia in 5% of LGLL cases [1,60,61,62]. As the underlying mechanism involves humoral immunity against mature erythrocytes, a diagnostic suspect of AIHA may be confirmed through the direct Coombs test (DCT). BM biopsy, instead, is required to confirm PRCA, reported in approximately one-third of T-LGLL patients presenting with isolated anemia [53]. A diagnosis of PRCA can be based on the evidence of a marked decrease or the absence of erythroid precursors and a lower reticulocyte percentage (at least <1%) [63]. No significant difference in terms of overall survival was observed in T-LGLL patients, irrespective of the presence of PRCA, suggesting that anemia, regardless of its pathogenesis, may represent an epiphenomenon of LGLL [53].

The detection of anti-platelet autoantibodies can instead suggest a diagnosis of ITP, reported in approximately 4% of LGLL cases [64]. It is noteworthy that a potential contribution of hypersplenism, which is a frequent feature of LGLL patients [50], cannot be excluded in thrombocytopenic cases, although it does not seem to correlate to the severity of cytopenia(s) [65]. LGLL-related amegakaryocytic thrombocytopenia (AMT) has also been reported [66,67] with mechanisms of immune mimicry towards the megakaryocytic lineage as potential pathobiological routes.

## 7. The Origin of Cytopenia(s): Is the LGL Clonal Expansion the Culprit?

In the context of chronic unexplained cytopenia, the diagnostic detection of an underlying LGL clonal expansion has probably been underestimated. On the other hand, pointing at the LGL clone as the main or unique cause of cytopenia would be reductive, as the association of these two entities, LGL clones and cytopenia, does not lead to a unique nosological entity. The diagnostic process is likely to be nuanced by the evidence of concurrent LGL clonal expansion in association with other hematological conditions, such as bone marrow failure syndromes (BMFS) and clonal myeloid diseases, which are all able, per se, to explain the presence of cytopenia [68].

Furthermore, increasing evidence has highlighted the frequent concurrence of LGL clones in the setting of conditions affecting the myeloid compartment, from clonal hematopoiesis of the indeterminate potential (CHIP) to myelodysplastic neoplasms/acute myeloid leukemia (MDS/AML) [5,69]. 

**Figure 2:**
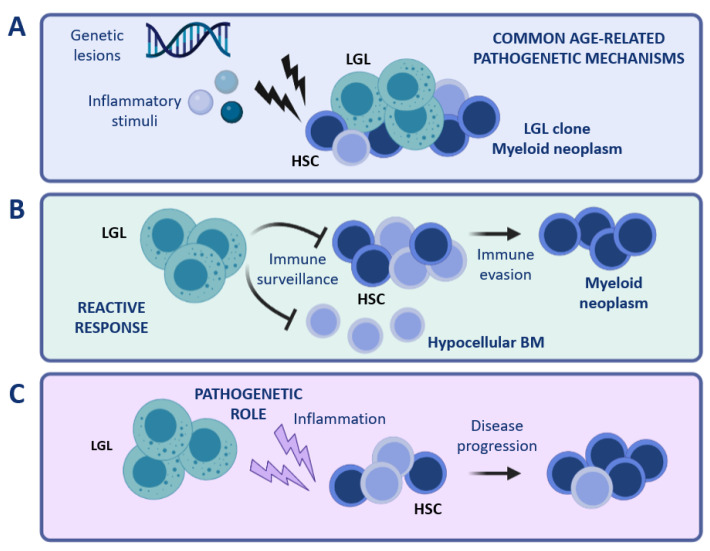
Coexistence of LGL and myeloid clones. Different scenarios can be hypothesized to explain the peculiar concurrence of LGL clones with myeloid diseases. (**A**) Common age-related mechanisms occurring in the BM compartment (for instance genetic lesions or inflammatory stimuli) might be involved in the pathogenesis of both conditions, i.e., LGL clonal expansion and myeloid disease. (**B**) LGL expansion might represent an immune reaction to the presence of aberrant HSCs. (**C**) A cytotoxic LGL clone might exert a pathogenetic role, causing damage in the myeloid compartment and favoring the development of a myeloid disease. Note: BM, bone marrow; HSC, hematopoietic stem cells; LGL, large granular lymphocytes. The figure was created with Biorender.com.

One could speculate that such peculiar associations may represent an extreme condition caused by common age-related pathogenetic mechanisms, such as common inciting stimuli, inflammatory environment, and mutational stress. (Figure 2A). With respect to this hypothesis, the finding of a shared sero-reactivity against a human T-cell leukemia virus epitope (i.e., BA21) in both LGLL and BMFS patients suggests that these diseases might share a common pathogenesis [70]. Regarding the occurrence of common genetic lesions, concurrent *STAT3*, *DNMT3A,* and *TET2* mutations were found in a patient with concomitant T-LGLL and CHIP [71]. Moreover, a recent study showed that somatic *TET2* mutations were shared by myeloid and NK cells in 3 out of 4 NK-LGLL cases, further supporting the hypothesis of common driver pathogenetic mechanisms [41].

A second possible scenario is that LGL clonal expansion might constitute a reactive phenomenon involved in tumor surveillance (Figure 2B). Under certain circumstances, the LGL-mediated immune surveillance might lead to an excessive eradication of BM cells, resulting in a hypocellular BM. Otherwise, immune evasion mechanisms might favor the establishment of a myeloid disease, which may develop simultaneously with the persistence of an expanded cytotoxic LGL clone.

Vice versa, clonal LGL expansions might have a causative role in the pathophysiology of myeloid diseases (Figure 2C). In this regard, inflammatory cytokines and soluble factors involved in the activation of different signaling pathways (such as IL-6, IL-15, and soluble Fas Ligand), produced and released by LGL cells [72], might exert chronic inflammatory pressure on BM HSC, resulting in persistent immune deregulation. In support of this latest hypothesis, we recently reported the association of clonal T cell expansions in high-risk hypoplastic MDS (MDS-h) patients [73].

The development of cytopenia can be the final result of a wide spectrum of alterations, occurring either in the BM compartment, at the level of myeloid precursors, or involving mature cells in peripheral sites. In some instances, the pathogenesis of cytopenia can also be multifactorial. In this context, it is still difficult to predict whether the LGL clone represents a pathogenetic determinant or instead a coincidental finding. Further investigations are needed to finally clarify the nature of such immune alterations.

## 8. LGL Clones as a Clue in the Differential Diagnosis of Cytopenic Patients

**Figure 3:**
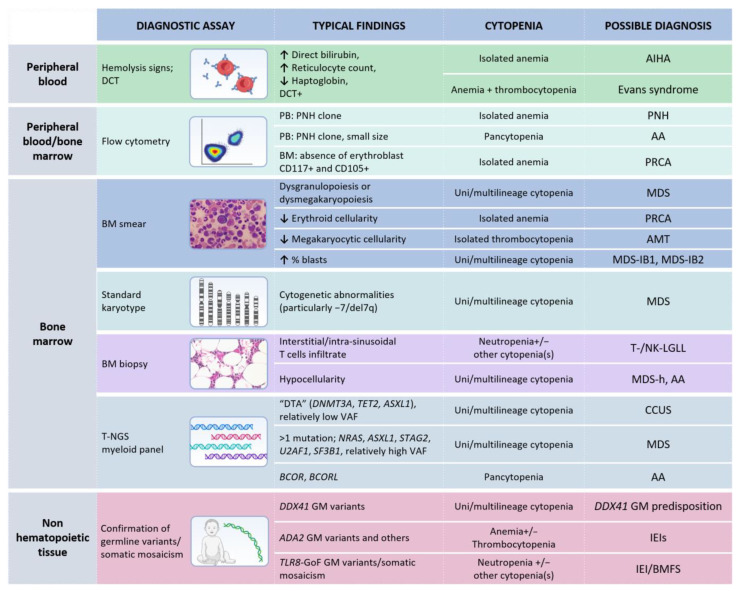
Diagnostic analyses recommended in case of unexplained cytopenia(s) and evidence of T- or NK-LGL clonal expansion. Note: Each test represents a mere hint towards a diagnostic definition and it is not enough for establishing a precise diagnosis. Germline tissue is intended as non-hematopoietic tissue (such as skin fibroblasts, nails, or buccal swab, according to local practice). MDS, when a specific subgroup is not specified, requires a specific diagnostic work-up in order to define features necessary for classification, according to the fifth WHO classification [20]. Abbreviations: AMT, amegakaryocytic thrombocytopenia; AA, aplastic anemia; BMFS, bone marrow failure syndromes; GoF, Gain of Function CCUS, clonal cytopenia(s) of undetermined significance; DTA, *DNMT3A-TET2-ASXL1*; DCT, direct Coombs test; GM, germline; IEI(s), inborn errors of immunity; LGLL, large granular lymphocytic leukemia; MDS, myelodysplastic neoplasms; MDS-h, hypoplastic MDS; MDS-IB, MDS with increased blasts; PNH, paroxysmal nocturnal hemoglobinuria; PB, peripheral blood; PRCA, pure red cell aplasia; t-NGS, targeted next generation sequencing; VAF, variant allele frequency. This arrow (↑) indicates an increase, while the other one (↓) a decrease. The table was created with Biorender.com.

In the landscape of BMFS, LGL clonal expansion may be considered as an additional clue for diagnostic purposes, rather as than the culprit of the cytopenia(s). In this regard, a multi-step approach is required to properly establish a diagnosis. Figure 3 provides an integrated view of the diagnostic procedures that should be considered according to the clinical setting and the available molecular data. 

### 8.1. Bone Marrow Smear and Biopsy

An evaluation of the BM morphology could provide immediate information. Dysgranulopoiesis and dysmegakaryopoiesis, with evidence of dysplasia in at least 10% of granulocytic precursors and megakaryocytes, point towards myelodysplastic neoplasms (MDS), in which cytopenia represents a condition for the diagnosis [74,75]. Dyserythropoiesis (even if isolated) is, instead, a less univocal feature, considering the overlap with the aplastic anemia picture [76]. Conversely, a deep reduction of the erythroid precursors in patients with both anemia and an LGL clonal expansion could support PRCA as the dominant disease entity [63].

Focusing on BM populations, an increase in BM blasts over 5–9% or 10–19% may support the diagnosis of MDS-IB1 and MDS-IB2, respectively [20]. In addition, BM biopsy, which is often underestimated and is considered as a complementary procedure, is able to clarify some nuanced features, such as BM cellularity. For the first time, the fifth edition of the WHO classification has recognized hypoplastic MDS (MDS-h) as a distinct clinical entity, defined according to an age-adjusted BM cellularity ≤25%. The relevance in distinguishing this rare subgroup of MDS is related to their frequent overlap with LGL clonal expansions, suggesting the involvement of peculiar immune mechanisms in disease pathogenesis and prognosis [73,77].

### 8.2. Bone Marrow/Peripheral Blood Flow Cytometry

Some currently available FC scores, such as the Ogata and Red scores, are helpful tools to support a diagnosis of MDS. Although not sufficient for MDS diagnosis, by integrating the information obtained from BM morphologic evaluation, these scores may help in defining a diagnosis of MDS as opposed to non-clonal cytopenia [78,79].

Concerning differential diagnosis with PRCA, FC is able to prove the absence of CD117+ and CD105+ erythroblasts in BM samples, which is a typical feature of PRCA, together with evidence of a severe reduction/absence of proerythroblasts; despite its diagnostic relevance, this approach is not currently standardized and PRCA remains a diagnosis by exclusion [63,80].

FC analyses of PB samples are also essential for the diagnostic work-up of paroxysmal nocturnal hemoglobinuria (PNH) clones [81]. The size of PNH clones, along with comprehensive BM characterization and clinical features, can guide towards PNH diagnosis, a condition in which hemolytic anemia is complement-mediated. Otherwise, in the context of BMFS patients usually have smaller, subclinical PNH clones, defined as an accompanying finding [82]. 

### 8.3. Bone Marrow Conventional Karyotyping and Myeloid Gene Mutation Analysis 

Considering the recurrence of LGL clones in the landscape of BMFS, conventional karyotyping is helpful for clarifying the cause of ineffective hematopoiesis. Cytogenetic abnormalities in AA are rare, while their incidence increases in MDS-h and even more in normo/hyperplastic MDS. Although there are no univocal abnormalities associated with a precise diagnosis, monosomy 7 and 7q deletion are consistent with MDS rather than AA [83].

Targeted next generation sequencing (t-NGS) techniques applied to the myeloid compartment have furthered our understanding of the genetic background in which LGL clones can emerge. Especially when BM dysplasia is the only additional finding in unexplained cytopenia(s) cases (without blast increase nor any cytogenetic abnormalities) a proper MDS diagnosis may be challenging, particularly for younger patients with hypocellular BM, where a correct differential diagnosis with other BMFS must be made. NGS could provide some additional information for diagnostic guidance, as over 90% of patients with myeloid diseases have been shown to present somatic mutations [84]. The occurrence of mutations in *DNMT3A*, *TET2,* and *ASXL1* genes in patients with LGLL has also been reported, suggesting their coexistence with CHIP or, at least in the presence of cytopenia(s), with clonal cytopenia of undetermined significance (CCUS) [5]. Clonal burden in terms of variant allele frequency (VAF) is typically lower in CCUS than in frank MDS [85]. However, typical CHIP mutations present with a higher clonal burden in LGLL compared with healthy individuals with CHIP, perhaps because of the expression of a more advanced disease status [5]. Accordingly, even in cases not fulfilling LGLL diagnostic criteria, the joint finding of cytopenia(s) and myeloid mutations, in the absence of BM dysplasia, might be ascribed to CCUS.

High-VAF mutations and the detection of multiple genetic variants in myeloid genes is highly indicative for an MDS diagnosis. Of note, different studies have shown that the co-occurrence of MDS with LGL clones could be related to the recurrence of some mutated genes, such as *NRAS* [86], *ASXL1*, *STAG2* [87], and *U2AF1*, while some disease-defining genes, such as *SF3B1*, are mutated equally in MDS/LGL and MDS alone [69]. *BCOR/BCORL1* mutations are common in aplastic anemia patients [88,89]; their presence in LGLL without evidence of any other hematological disorders suggest that they may occur both in myeloid and lymphoid cells [5].

### 8.4. The Landscape of Germline Immune-Hematological Disorders

The widespread availability of large-scale genomic sequencing has recently promoted the investigation of germline variants associated with non-syndromic BMFS cases, which can occur without any other clinical signs except for cytopenia(s). The current knowledge of these variants is indeed leading to a reassessment of the boundaries initially conceived for some phenotypes. This is the case of *DDX41*, originally classified as one of the most commonly mutated genes in familial myeloid neoplasms, whose variants are now also recognized in hematolymphoid neoplasms, such as T-LGLL and multiple myeloma [90].

Mutational analysis of this inherited predisposing gene should be included in the diagnostic routine, considering that *DDX41* variants are not associated with distinctive pathologic features and the median age of disease onset overlaps with de novo cases.

Especially in younger patients, LGL clonal expansion and cytopenia could suggest other peculiar inherited disorders. In particular, inborn errors of immunity (IEIs) may confer susceptibility to chronic antigen exposure that lead to the clonal proliferation of lymphoid cells. T-LGL clonal expansions (sometimes fulfilling T-LGLL criteria) have been associated with germline mutations in *ADA2* [91], *CARD11* [92], and *LRBA* [93]. The recent discovery of *TLR8*-GoF variants confirmed the importance of investigating genetic variants in IEIs, which are not necessarily defined based on their germline nature. *TLR8*-GoF variants, indeed, can occur alternatively as germline events or as postzygotic ones, leading to somatic mosaicism [94]. These variants could determine chronic neutropenia as the dominant cytopenia, accompanied by an immune dysregulation phenotype and T-cell expansion in BM. The combination of defects in immune function and BM cell production place this disorder at the interface between two distinct landscapes of rare diseases, BMFS and IEIs [95]. In other cases, LGLL was reported in common variable immunodeficiency (CVID) [96], Hyper-IgM syndrome [97], and even acquired conditions such as Good syndrome [4], oftentimes without a proper genetic diagnosis. A diagnostic work-up in IEIs patients frequently includes in-depth T-cell subset characterization by FC, which might lead to the identification of an LGL expansion [98]. In these cases, assessment of clonality could be crucial to obtain new insights in how lymphoproliferation may occur in the clinical setting on IEIs. Considering that primary immune regulatory disorders (PIRDs) mainly present with autoimmune cytopenias as the first clinical sign [99], cytopenia(s) and LGL clonal expansions may possibly represent two sides of the same coin in the field of primary immunodeficiencies.

## 9. Conclusions

LGL clonal expansion can be detected in a wide spectrum of conditions, including LGLL, a rare and heterogeneous hematological disease characterized by chronic cytopenias as the most frequent clinical manifestation. Despite the efforts in improving molecular diagnostics, the boundaries between LGLL, intended as an independent disease entity, and LGL clonal expansion still lie in ill-defined diagnostic shadowlands. Of note, LGL clonal expansions might acquire different clinical relevance according to the setting in which they occur, ranging from both hematological to autoimmune disorders and immunodeficiencies. In some cases, LGL clones have been associated with worse survival outcomes, being recognized as being responsible for complex clinical phenotypes. However, whether LGL clones represent a reactive immune response or, vice versa, they are part of a pathogenetic mechanism that still remains a matter of debate. Beyond their still unclarified pathophysiologic role, LGL expansions should be considered an additional clue to guide clinicians in the landscape of cytopenic disorders. As for their clinical implications, the investigation of the biological significance of T- and NK-LGL clonal expansions might be crucial, especially in the setting of unexplained chronic cytopenias, as their detection and characterization could provide relevant prognostic information to properly inform therapeutic choices.

## Data Availability

The data presented in this study are available in this article.
